# Vitamin D in Wild and Farmed Atlantic Salmon (*Salmo Salar*)—What Do We Know?

**DOI:** 10.3390/nu11050982

**Published:** 2019-04-29

**Authors:** Jette Jakobsen, Cat Smith, Anette Bysted, Kevin D. Cashman

**Affiliations:** 1 National Food Institute, Technical University of Denmark, 2800 Lyngby, Denmark; anby@food.dtu.dk; 2 Bantry Marine Research Station, Gearhies, Bantry, P75 AX07 Cork, Ireland; catsmith85@gmail.com; 3 Cork Centre for Vitamin D and Nutrition Research, School of Food and Nutritional Sciences, University College Cork, T12 Y337 Cork, Ireland; k.cashman@ucc.ie

**Keywords:** vitamin D, vitamin D_3_, wild, farmed, aquaculture, salmon, salmonids, *Salmo salar*

## Abstract

Salmon have been widely publicized as a good dietary source of vitamin D, but recent data points to large variation in vitamin D content and differences between wild and farmed salmon. We aimed to: (1) investigate the content of vitamin D in Atlantic salmon (*Salmo salar*) in wild species caught in two different waters, (2) perform a 12-week feeding trial in farmed *Salmo salar* with 270–1440 µg vitamin D_3_/kg feed (4–20 times maximum level in the EU) and (3) conduct a review for the published data on the content of vitamin D in salmonids. Content of vitamin D_3_ in the fillet from wild salmon caught in the Baltic Sea and the North Sea was significantly different (*p* < 0.05), being 18.5 ± 4.6 µg/100 g and 9.4 ± 1.9 µg/100 g, respectively. In the farmed salmon the content ranged from 2.9 ± 0.7 µg vitamin D_3_/100 g to 9.5 ± 0.7 µg vitamin D_3_/100 g. Data from 2018 shows that farmed salmon contained 2.3–7.3 µg vitamin D_3_/100 g. Information on the content of vitamin D in wild and farmed salmonids is very limited, which calls for further research to ensure a sustainable production of salmon with adequate vitamin D.

## 1. Introduction

There has been major interest in recent decades in the possible role of the low vitamin D levels in the risk of various non-skeletal diseases, such as cardiovascular disease, diabetes, certain types of cancer, infectious disease, or other autoimmune and inflammatory disease, as well as to its more accepted role in metabolic bone disease risk [1,2]. The two modes of obtaining vitamin D are through synthesis in the skin, triggered by sun exposure, and through diet. There are several environmental factors which determine whether or not sunlight of sufficient strength is available for the cutaneous synthesis of vitamin D, including latitude and prevailing weather conditions [1]. In recent years there has been an increased awareness of the dangers associated with sun-exposure and the damage it can cause to health. This has resulted in an increase in precautionary habits such as sun avoidance, covering of skin and application of protective creams and, thus, causing a decrease in dermal vitamin D synthesis via sun exposure [2]. At latitudes above 35°, the cutaneous production of vitamin only occurs in one part of the year, and the time without skin synthesis of vitamin D, referred to as the “vitamin D winter”, had been estimated to range from being largely absent in the southernmost Europe, to lasting for as long as 7 or 8 months in the north of Norway [3]. The prevalence of vitamin D deficiency (serum 25 hydroxyvitamin D (25(OH)D) <50 nmol/L) in Europe was recently estimated to be 40%, based on standardised 25(OH)D data from 18 key representative studies of European older adults, adults, teenagers and children, which encompassed almost 56,000 people [4]. The recommended dietary intake of vitamin D is 10–20 µg/day [5,6,7,8]. Current dietary intakes of vitamin D have been estimated to be about 3–7 μg/day, depending on diet, age, sex and fortification practices [9].

Food that is considered to be a good source of vitamin D include fatty fish, cod liver oil, and eggs [10]. Salmon as a fatty fish is generally regarded as having high vitamin D content. Wild salmon is the name commonly used to refer to species in the Salmonidae family, with *Salmo salar (S. salar*) being the salmon living in the Atlantic Ocean, and the salmon of the *Oncorhynchus sp* in the Pacific Ocean. The latter includes the seven species—rainbow trout (*Oncorhynchus mykiss; O. mykiss*), chum salmon (*Oncorhynchus keta*), chinook salmon (*Oncorhynchus tshawytscha*), coho salmon (*Oncorhynchus kisutch*), masu (*Oncorhynchus masou*), pink salmon *Oncorhynchus Gorbuscha* and sockeye salmon (*Oncorhynchus nerka*) [11]. In the EU, the consumption of salmonids was in total 1,338,514 tonnes in 2016, which covered 4.6% of wild species and 95.6% of farmed species [12]. Worldwide salmon and trout is the largest single fish commodity by value (18.1%) but by weight it is about 7.4%; of this, approximately 67% is *S. salar* and 33% is *O. mykiss* [13]. The vitamin D content in wild salmon has been reported as 8–55 µg vitamin D/100 g [14,15,16]. Of note, however, the vitamin D_3_ content in farmed salmon have been reported to be in the range 4.2–10 µg/100 g [15,16,17,18,19], and in Food Composition Databases, a content of 11 µg vitamin D/100 g was observed [20]. This difference in the vitamin D content of wild and farmed salmon might relate to their dietary supply of vitamin, which seemed to be more important than the endogenous synthesis in fish [21,22]. Wild salmon in the North Atlantic are likely to be getting their dietary supply of vitamin D through consumption of crustaceans, e.g., amphipods, euphausiids and shrimps, and to a lesser extent, fish, e.g., pearlsides and lanternfish, [23], whereas farmed salmon get their vitamin D via pelleted feed, i.e., fishmeal, vegetable oil, minerals and vitamins. In the EU, the maximum allowed addition of vitamin D_3_ in fish feed is 75 µg/kg [24,25,26]. The EFSA FEEDAP Panel has recently stated that salmonids are highly tolerant of vitamin D_3_ and has estimated a safe upper dietary level of 25 mg vitamin D_3_/kg feed for salmonids, and suggest that 1,500 µg/kg in the feed would lead to safe fish products for the consumer [27].

Information from feeding trials in salmonids is limited. Previous studies in *S. salar* with the initial weight of 177 g and 170 mg [28,29] and in *O. mykiss* with an initial weight of 500 g [30] have been conducted. In *S. salar*, an increased content of vitamin D_3_ in either the fillet (up to 210 µg vitamin D_3_/100 g) or the whole fish (up to 650 µg vitamin D_3_/100 g;) were reported, with increasing content of vitamin D in the feed (40–28,000 µg/kg feed and 200–57,000 µg/kg feed, respectively). Of note, there was no evidence of detrimental effects with the increasing feed in vitamin D levels [28,29]. In contrast, in *O. mykiss*, the content of vitamin D_3_ in the fillet was unaffected by the increasing vitamin D_3_ in the feed (9–539 µg vitamin D_3_/kg feed) [30].

The objectives in this study was to test the following hypotheses: (1) vitamin D content in wild Atlantic salmon (*S. salar*) is independent of the catch area, (2) increased levels of vitamin D_3_ in fishfeed, at high levels, will produce salmon fillet with similar levels as wild salmon. Wild *S. salar* was caught in two different waters (Baltic Sea and North Sea), and a feeding trial in *S.salar,* at an initial weight of 1 kg, with 4 levels of vitamin D_3_ (270–57,000 µg/kg feed) was run for 12 weeks. A third objective was to conduct a review on the data published on the content of vitamin D in wild and farmed, raw salmonids, *S. salar* and *Oncorhynchus sp.,* against which our new data could be bench-marked.

## 2. Materials and Method

### 2.1. Wild Salmon

#### Sampling

Five Atlantic salmon (3.5–4.7 kg gutted weight) were caught in the Baltic Sea (catch area FAO-27, 3D) in March 2014, and five Atlantic salmon (3.3–7.1 kg gutted weight) from the North Sea were caught in Skjern Å, Jutland, Denmark (55°N, 12°E) in June 2016. From each salmon, one of the fillets was separated for analysis.

### 2.2. Farmed Salmon

#### 2.2.1. Experimental Procedure

The vitamin D_3_-enriched feed for salmon was supplied by Sparos Lda, Olhão, Portugal with a content of nutrients in accordance with commercial diets for a salmon of this size. Detailed information on the feed is provided in Table S1 in the Supplementary Material. The intended vitamin D levels within the feeding trial were 75 µg/kg and were also five-, ten-, and twenty-times this level. Subsequent analysis of the feed showed the actual levels differed from what was expected, being about 270 µg/kg, 590 µg/kg, 890 µg/kg and 1440 µg/kg of vitamin D_3_. The salmon were fed 1% body weight/day.

In October 2014, the salmon (weighing approximately 1 kg) for the feeding trial were obtained from Murphy’s Irish Seafood, South-West Ireland. Forty salmon were put into each of four 8000 L tanks at Bantry Marine Research Station, Cork, Ireland. The fish were acclimatised to the tanks for two weeks, prior to the start of the trial. The salmon receiving 590 µg vitamin D_3_/kg were given the correct feed, but transferred to the trial tanks two weeks later than the other three feeding groups, due to technical problems.

Each tank was supplied with filtered seawater and a steady supply of oxygen. Environmental parameters (dissolved oxygen concentration, salinity and temperature) were measured daily. The tanks were equipped with probes, which measured the oxygen levels every 10 min and automatically dosed oxygen, in order to keep the level at >100%. The natural salinity varied from approximately 33.3 ppt to 36.8 ppt, while the water temperature varied between 8.4–14.6 °C, as measured by the OxyGuard Handy Polaris (OxyGuard International, Farum, Denmark). Any behavioural changes were noted, this included incidences where the fish did not feed as voraciously as usual.

#### 2.2.2. Sampling

Ten individual salmon were randomly selected at day 0 of the trial to obtain weight and fillet vitamin D content at baseline. Subsequently, ten individual salmon were randomly selected from each tank after four, eight and twelve weeks of the feeding trial, to obtain the weight to estimate the growth performance and the content of vitamin D in the fillet. This involved euthanising with tricaine mesylate, before measuring the weight, followed by removing one of the fillets. Samples were stored at −18 °C, until the end of the trial, where they were sent to the Technical University of Denmark (DTU) for vitamin D analysis.

### 2.3. Review for Data on Vitamin D in Salmonids

A literature search up to February 2019 was performed with the search criteria of (“vitamin d*” AND (fish OR salmon OR salmonid OR trout OR salmonidae)) AND (food) NOT (breastfeeding OR status OR deficiency OR “human milk” OR “mothers milk”) in DTU FindIT [31]. In addition reports documenting the data in food composition databases were included. Results provided in IU were multiplied 0.025, in order to be converted to µg.

### 2.4. Laboratory Analyses

#### 2.4.1. Vitamin D in Salmon Fillets and Fishfeed

The amounts of vitamin D_3_, 25-hydroxyvitamin D_3_ (25(OH)D_3_), vitamin D_2_ and 25-hydroxyvitamin D_2_ were detected and quantified by the use of liquid chromatography, combined with triple quadrupole mass spectrometry and electrospray ionisation (Agilent 1200 Series and Agilent 6470, Agilent Technologies, Santa Clara, CA, USA) [32,33]. The homogenized samples were stored in plastic bags at maximum −20 °C, until analysis within 6 months. The limit of quantification (LOQ) for vitamin D (D_3_ and D_2_) and for 25-hydroxyvitamin D were (D_3_/D_2_) 0.05 µg/100 g and 0.03 µg/100 g, respectively, while the precision for both was <10% (CV). The in-house control salmon included in all analytical series, documented the correctness during the analyses in the whole study, performed over a period of 2.5 years. The in-house control salmon contained 15.0 µg vitamin D_3_/100 g ± 6.8% and 0.28 µg 25-hydroxyvitamin D_3_/100 g ± 10%. All analyses performed were accredited according to ISO17025.

#### 2.4.2. Fat Content in Wild Salmon

The content of fat in the fish was determined gravimetrically by a modified Schmid-Bondzynski-Ratslaff (SBR) procedure [34].

### 2.5. Statistical Analysis

T-test was used to test for differences between the two groups of wild salmon. One- and Two-way-Analysis of Variance (ANOVA) followed by Tukey’s HSD post hoc tests were used to test for differences between the groups, within the farmed salmon feeding trial, and the Pearson correlation coefficient was used to identify the association between the two variables (Microsoft, Excel 2010, Washington, DC, USA). A *p*-value < 0.05 was classified as a significant difference. All results are presented as mean ± standard deviation (SD). Conversion rate was calculated as the vitamin D content in a fillet (µg/kg) of salmon divided by vitamin D content in the feed (µg/kg).

## 3. Results

### 3.1. Wild Salmon

**Figure 1:**
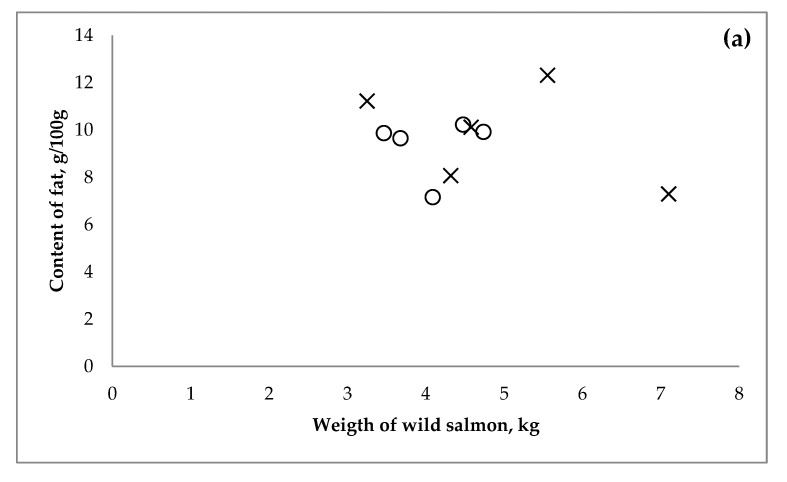

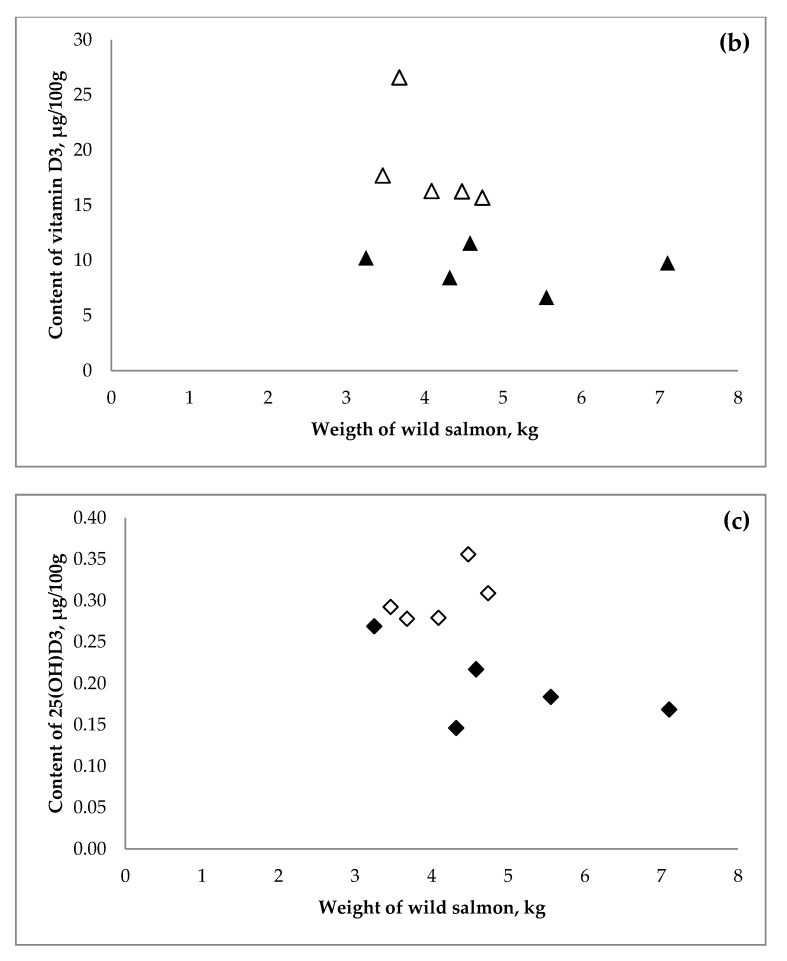
(**a**) Fat content in fillet as a function of weight of the wild Atlantic salmon (*Salmo salar*). Fat in salmon from the Baltic Sea (○) and from the North Sea (x). (**b**) Vitamin D_3_ in fillet as a function of weight of the wild Atlantic salmon (*Salmo salar*). Vitamin D_3_ in salmon from the Baltic Sea (Δ) and the North Sea (▲). (**c**) 25(OH)D_3_ content of fillet as a function of the weight of the *Salmo salar*. 25(OH)D_3_ in salmon from the Baltic Sea (◊) and the North Sea (♦).

The results for the content of fat, vitamin D_3_ and 25(OH)D_3_, as a function of weight of the wild salmon are shown in Figure 1. No significant difference were observed in either weight (4.1 ± 0.5 kg vs. 5.0 ± 1.5 kg, respectively) or content of fat (9.4 ± 1.3 g/100 g vs. 9.8 ± 2.1 g/100 g, respectively) between the group of salmon from the Baltic Sea and the North Sea (*p* > 0.3 for both), but there was a significant difference (*p* < 0.05) in the content of vitamin D_3_ (18.5 ± 4.6 µg/100 g vs. 9.4 ± 1.9 µg/100 g, respectively) and 25(OH)D_3_ (0.30 ± 0.03 µg/100 g and 0.20 ± 0.05 µg/100 g, respectively). Furthermore, the Pearson correlation coefficient between the content of vitamin D_3_ and fat within the two groups was <0.3.

### 3.2. Farmed Salmon

#### 3.2.1. Performance of Fish

**Figure 2:**
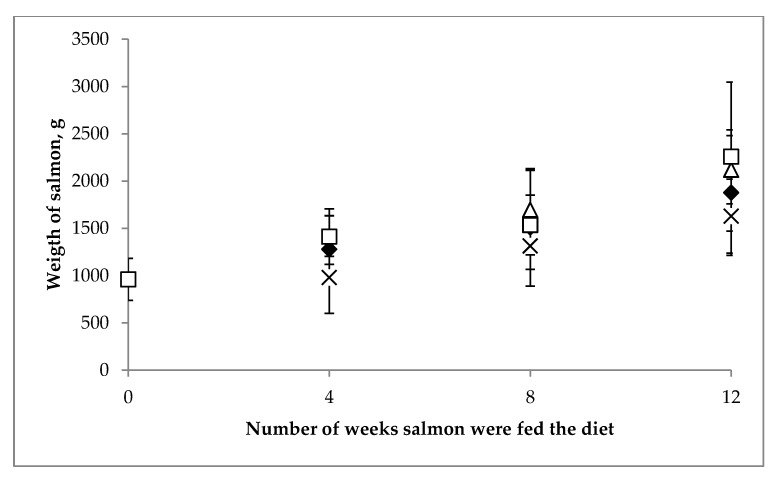
Growth performance shown as weight (x ± SD) in grams for farmed *Salmo salar* (*n* = 10) at baseline, week 4, 8 and 12, in the four feeding groups receiving vitamin D at the dietary level of 270 µg/kg (♦), 590 µg/kg (x), 890 µg/kg (Δ) and 1440 µg/kg (□).

The increase in salmon biomass in each of the four feeding groups at the four times points are presented in Figure 2. There was a significant increase in weight in all four groups (*p* < 0.001), over the 12 weeks of the trial, with no significant difference (*p* = 0.10) in the final weight of salmon, across the 4 treatment groups. Furthermore, no physical or behavioural abnormalities were evident in the salmon, during the trial, and no mortalities were observed.

#### 3.2.2. Vitamin D Content of Farmed Salmon

**Figure 3:**
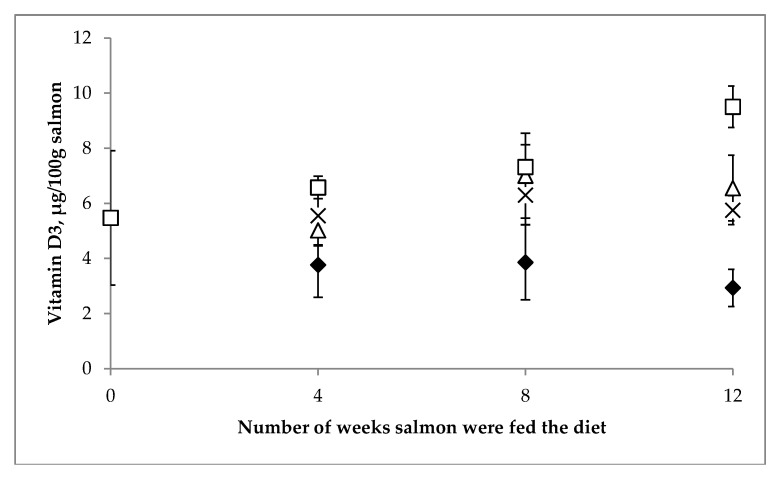
Mean vitamin D_3_ (µg/100 g) in fillet of farmed *Salmo salar* (*n* = 5) at baseline, week 4, week 8 and week 12, in the four feeding groups receiving vitamin D, at the dietary level of 270 µg/kg (♦), 590 µg/kg (x), 890 µg/kg (Δ) and 1440 µg/kg (□). Error bars representing the SD around the means.

The mean content of vitamin D_3_ in salmon fillets from each of the four treatment groups are shown in Figure 3. Two-way ANOVA showed that there was a significant time (i.e., weeks of feeding) main effect (*p* < 0.01), a significant treatment group main effect (*p* < 0.001), as well as a significant interaction between these two main effects (*p* < 0.05). Specific values are provided in Table S2 in the Supplementary Material. After 12 weeks of feeding, a significant difference in the vitamin D_3_ content of fillet was evident between the four feeding levels (*p* < 0.001), such that the content in the group receiving 270 µg/kg was lowest (2.9 ± 0.7 µg/100 g), while it was highest in the salmon receiving 1,440 µg/kg (9.5 ± 0.7 µg/100 g) and intermediate in the groups receiving 590 and 890 µg/kg (5.8 ± 0.5 and 6.6 ± 1.2 µg/100 g, respectively).

Two-way ANOVA showed no significant effect of time or treatment group on mean 25(OH)D_3_ content of the salmon fillet; the mean (± SD) content of all salmon at the endpoint was 0.14 ± 0.05 µg/100 g. The vitamin D_2_ and 25-vitamin D_2_ content of all salmon were below the LOQ.

The conversion rate from µg vitamin D_3_/kg feed to µg vitamin D_3_/kg fish fillet decreased from 0.11, 0.10, 0.07 to 0.07 for feeding with 270, 590, 890 and 1,440 µg vitamin D_3_/kg feed, respectively.

### 3.3. Published Data on Vitamin D in Wild and Farmed Salmonids

A search of the existing literature showed that the data on vitamin D are limited, and is even more limited for data which provide a thorough description of the salmon analysed. This is especially the case for wild salmon, which might either be from the Atlantic Ocean or the Pacific Ocean. For example, Chen et al. [35] reported the results for the content of wild salmon to be 24.5 ± 2.2 µg/100 g, however no information was provided for the origin of the twenty wild salmon collected and analysed. Moreover, data in food databases were only included if the data had an original reference. For this reason, the value of 11 µg vitamin D/100 g farmed salmon has not been included [20]. Results reporting the content of vitamin D_3_ and 25(OH)D_3_ in wild and farmed salmonids, provided with the sufficient identification of the fish and collection methods, are collated and presented in Table 1 and Table 2, respectively.

## 4. Discussion

Data on vitamin D in wild salmon are limited, which is likely related to the challenges of vitamin D quantification in foods, including it being a time-consuming and also very expensive analysis. There is very old data on vitamin D in three salmons, caught in the period 1953–1980, which reported a content of 8, 30 and 55 µg vitamin D/100 g, respectively [14]. However, those data are based on analysis of salmon using the biological assay for vitamin D, which estimated the total vitamin D activity to eradicate rickets in rats and which did not discriminate between the different vitamin D active compounds. More recently, salmon (*n* = 10) caught in the Baltic Sea showed a high, but variable, vitamin D_3_ content at 26.5 ± 5.6 µg/100 g [16], while two composite samples (*n* = 2×10) from the Atlantic Ocean near Ireland had a mean content of 9.6 µg/100 g [15]. The respective content of fat in salmon from these two studies was 12.1% and 16.5%. The results from our present study show differences in vitamin D content of a similar magnitude, between salmon caught in the Baltic Sea (in March) and from the North Sea (in June) i.e., 18.5 ± 4.6 and 9.4 ± 1.9 µg vitamin D_3_/100 g, respectively (*p* < 0.05). Our two groups of salmon had a content of fat at 9–10%. These data highlight that the comparison between the vitamin D content of wild salmon needs to be interpreted carefully, as the time and location of sampling might influence the content of vitamin D_3_. This might also contribute to the reported extreme variation of vitamin D in wild salmon, over a period of more than 50 years (8–55 µg/100 g). Potentially, the use of the average vitamin D content of the three salmons caught from 1953–1980 (i.e., 30 µg vitamin D/100 g) in the Danish Food Databank might have led to an overstatement that wild salmon (*Salmo salar*) in general has an extremely high content of vitamin D.

The present work showed that the vitamin D content of fillets from farmed salmon at baseline of the feeding trial (mean, 5.5 µg/100 g) was lower than that of fillets from the wild salmon. The farmed fish were much lighter (~1 kg), compared to the wild salmon (3–7 kg). However, in an unpublished study, we have shown that in a batch of salmon (*n* = 13) from the Baltic Sea there was no association between the weight of salmon (2–12 kg) and the content of vitamin D_3_ (11.4–21.9 µg/100 g) [40]. Neither did we find an association between vitamin D_3_ and weight in our wild salmon, in the present work (3.3–7.1 kg).

Increasing the vitamin D level in the feed of farmed fish, as a strategy of increasing the fillet vitamin D content, was tested in the present feeding trial of farmed salmon (with the initial weight of ~1 kg). Of note, in terms of the safety of this approach, there were no significant differences recorded in the final weight and mortality rate, which is in agreement with the findings of the other vitamin D feeding studies of salmon (initial weight of ~170 mg–2 kg) [28,29,41]. Graff et al. [41] recently reported that feeding salmon (with an initial weight of 2 kg) for 3–4 months with 230, 2600 and 2900 µg vitamin D_3_/kg, resulted in a content of vitamin D_3_ in the fillet, of approximately 9 µg/100 g, 35 µg/100 g, and 38 µg/100 g, respectively. In our study, we observed a significant increase in vitamin D content, following feeding of an additional vitamin D over 4, 8 or 12 weeks; however, the increments achieved were far less than those in the Graff et al. [41] trial. Even at the vitamin D_3_ addition level of 270 µg/kg feed (3.6 times higher than the current limit in the EU), we found a surprisingly low vitamin D content in the salmon fillet at 2.9 µg/100 g; only 30% of the level reported by Graff et al. [41], for salmon in the 230 µg/kg feeding group. While our maximum level of 9.5 µg vitamin D_3_/100 g fillet, achieved by feeding 1440 µg/kg, was similar to the wild salmon caught in the North Sea, it was, however, significantly lower than the wild salmon caught in the Baltic Sea.

In the above-mentioned studies, the conversion rate showed trend similar to our study—decreasing with an increase of vitamin D_3_ in the feeding, being 0.25–0.07 for the salmon with an initial weight of 177 g [28], and 0.39–0.13 for the salmon with an initial weight of 2 kg [41]. However, ours were at a lower level, being 0.11–0.07, and furthermore, we saw a huge variation within the feeding groups of salmon. This large variation between fish might have explained, at least in part, the lack of significant increase in the content of vitamin D_3_ in the salmon fillet arising from an increased vitamin D_3_ in the feed to 590 and 890 µg vitamin D_3_/kg feed. Much smaller variations have been reported from dose-related vitamin D-feeding trials in pigs, hens and cattle [42,43,44], which made it easier to identify significant differences. For example, in a dose-related vitamin D_3_ feeding trial in pigs, the analysis of cuts from 4 pigs was sufficient to see the significant differences between all four feeding group at 5 µg, 20 µg, 35 µg and 50 µg vitamin D_3_/kg feed [42].

We propose that the low vitamin D content of the fillets in the salmon in our group, given the lowest level of vitamin D_3_ as well as the huge difference observed in the conversion rate in the feeding trial conducted, could be caused by the huge natural variation in wild salmon. No genetic information was collected, but a study comparing the two strains of salmon showed a significant difference in growth performance, i.e., weight growth [45]. This might also highlight that biofortifying farmed salmon should include an initial analyses of vitamin D as a base in future breeding programs, especially as the content of vitamin D in wild salmon differs.

The data in Table 1 show the studies identified to provide the content of vitamin D_3_ in wild or farmed salmonids i.e., *S. salar* or *Oncorhynchus sp*, including the data obtained in the present study. The samples of the farmed *S.salar* analysed was sampled from 2002 up to 2018. Primarily these studies have included analyses of composite samples, and thus, did not contribute to any information on the variation. However, the mean from the individual studies varied from 5.8 to 11 µg vitamin D_3_/100 g. The most recent data were farmed salmon bought in Denmark in August 2018 (*n* = 12) which had a content between 2.3–7.3 µg vitamin D_3_/100 g fillet and had a mean content of 6.0 µg/100 g [18]. The farmed salmon originated from Norway, The Faroe Island and Scotland.

Due to the limited number of individual salmons analysed and the huge variation found in the individual studies, any trend analyses of the content of vitamin D_3_ in farmed salmon should be done with caution. That said, the data on farmed salmon produced in 2002–2003 showed a minimum of 4.2 µg vitamin D_3_/100 g, albeit in a composite sample of 5 salmons [15]. Thus, the minimum content for the individual salmon could possibly have the same level as observed in 2018 at just 2.3 µg vitamin D_3_/100 g [18]. For the farmed trout (*O. mykiss*) the information was from 1991 to 2015, and similar to the farmed salmon it was difficult to identify a trend, due to the different sampling strategy and analyses of single or pooled samples. Furthermore, we have no information on the feeding system, including the content of vitamin D_3_ in the earlier studies. However, there has been a marked increase in the use of plant-based ingredients in fish feed, including soy, peas, horse beans, grains and rapeseed oil [46]. Amount of non-marine oil and protein in Norwegian produced farmed salmon has increased from 10.6% in 1990, 35.4% in 2000, up to 58.6% in 2010 and 70.8% in 2013 [47]. The replacement of fish based feed to partly vegetable ingredients in fish feed has been identified by a decrease of the essential n-3 long-chain polyunsaturated fatty acids, eicosapentaenoic acid (EPA) and docosahexaenoic acid (DHA) in the salmon aquaculture in Scotland from 2006–2015 [48], and in aquacultured salmon marketed in Denmark 2007 and 2018 [18]. A decrease in content of vitamin D_3_ has been observed in fish feed marketed in Norway 2002–2017 [49,50], a decrease in the content of vitamin D_3_ in fish fillet is possible, but was not included or identified in these reports.

The strength of this study was that wild and farmed salmon was included and all analyses for vitamin D were run in the same ISO-accredited laboratory. The limitation was that sampling from both oceans and the feeding trial, was only performed once. Preferably all biological tests should be run in triplicates, but this was not feasible in the present work, due to the financial restrictions. Another implication is that it would have been valuable to include a larger number of salmons due to the huge variation observed. It should be noted that none of the other feeding trials reported was run in triplicates.

Besides vitamin D_3_, the vitamin D metabolite, 25(OH)D_3_ contributed to the total vitamin D activity. Only four studies included the quantitation of 25(OH)D_3_, see Table 2. In general the content was low, approximately 0.3–0.5 µg/100 g, but the most recent study, single analyses of the o12 samples of farmed *Salmo salar* found a mean of 0.7 µg/100 g and a maximum of 1.5 µg/100 g [18]. It should be stressed that no international consensus has been established to convert the content of 25(OH)D_3_ to vitamin D_3_. The latest review on this issue proposed a factor of 3 [51], while the sole human intervention study conducted as a cross-over, resulted in a factor of 1.5 [52].

The nutritional significance for the dietary intake of vitamin D from salmon could be calculated for the average European citizen, based on the estimations of production, import and export in the EU [12]. The optimal data to base these estimates on would be on data from dietary survey, which was available for Danes. For Danish fish-eaters, the average person has a mean intake of 16 g salmon per day, while the 95%-percentile has a daily intake of 51 g salmon. If the salmon eaten is the wild species, with the highest reported content, the dietary intake of vitamin D from salmon alone would be 3.5 µg/day and 13.5 µg/day, for the mean, and 95%-percentile, respectively. In contrast, if the most recent data for vitamin D in farmed salmon was used, the daily intake would be 1.0 µg and 2.9 µg, respectively [18,53]. This highlights how, if farmed species are produced with a lower content of vitamin D, it would have a significant negative impact on the dietary intake of vitamin D, even though the farmed salmonids would still contribute significantly to the overall dietary intake of vitamin D, because quantitatively, for many populations, this is <5 µg/day [9].

## 5. Conclusions

Salmonids cover the Atlantic salmon (*Salmo salar*) and the Pacific salmon (*Oncorhynchus* sp.). Content of vitamin D_3_ in the fillet from wild Atlantic salmon caught in the Baltic Sea was significantly higher than fillet from salmon caught in the North Sea, being 18.5 ± 4.6 µg/100 g, and 9.4 ± 1.9 µg/100 g, respectively. In the feeding trial with the farmed Atlantic salmon, the four groups fed 270–1440 µg vitamin D_3_/kg feed had a content in the salmon fillet which ranged from 2.9 ± 0.7 µg vitamin D_3_/100 g to 9.5±0.7 µg vitamin D_3_/100 g. The content of 25(OH)D_3_ in all samples was <0.4 µg/100 g. A review of the published studies which report the content of vitamin D in wild and farmed salmonids, showed very limited data, and even within the available data, huge variation in sampling and number of samples were included. Salmon is an excellent source for vitamin D, but these new results on the wild Atlantic salmon and from the feeding trial, using an excessive amount of vitamin D in the feed, calls for further research to minimize the variation in the farmed species, and to ensure a sustainable production of salmon with regards to vitamin D, for the benefit of the consumer.

## Figures and Tables

**Table 1:** Content of vitamin D_3_ (µg/100 g fillet) of Atlantic salmon (*Salmo salar*) and Pacific salmon (*Oncorhynchus sp.*) from 1953–2018, either wild or farmed. If published, sampling year, number of samples (*N*, either analysed in single or as composite samples), mean ± SD and range are included.

Year of	Salmon (*Salmo Salar*)						Salmon (*Oncorhynchus sp.*)						
Sampling	Wild			Farmed			Wild ^x^			Farmed ^y^			
	*N*	mean ± SD	Range	*N*	mean ± SD	Range	*N*	mean ± SD	Range	*N*	mean ± SD	Range	Ref
1953–1980	3	30 ± 24 ^a,c^	8–55										[14]
1991										9	13 ± 3	8–16	[36]
1993										* 2 × 10	7.6	7.3–7.8	[37]
1996										11	18.7 ± 3.8	10–23	[38]
2002–2003	* 2 × 10	9.6	8.4–10.7	* 4 × 10	7.6	4.2–9.1				* 4 × 20	8.1	3.8–10.7	[15]
2007 ^b^				24	6.2 ± 1.0 ^c^					12	9.3 ± 1.6 ^c^		[35]
2007–2008				* 20	10					* 12	6.9		[17]
2007–2008				* 2 × 10	6.7	6.5–7.0	* 2 × 10	14.2	12.7–15.6				[18]
2011										* 9	7.0		[39]
2014	5	18.5 ± 4.6	15.7–26.6										-
2015 ^b^	10	26.5 ± 5.6		10	5.9 ± 3.6					10	8.0 ± 3.4		[16]
2016 ^b^				3	5.8	3.6–8.2							[19]
2016	5	9.4 ± 1.9	6.7–11.6										-
2018				12	6.0 ± 1.4	2.3–7.3							[18]

* Composite samples: ^a^ Biological assay; ^b^ Publication year; ^x^ *O. gorbuscha* and *O. keta* and *O. sp.;* ^y^ *O. mykiss*; ”-“ this study; ^c^ The specific name not included in the reference.

**Table 2:** Content of 25-hydroxyvitamin D_3_ (µg/100 g fillet) of Atlantic salmon (*Salmo salar*) and Pacific salmon (*Oncorhynchus sp.*) from 2007–2018, either wild or farmed. If published, sampling year, number of samples (either analysed in single or as composite samples), mean ± SD and range are included.

Year of	Salmon (*Salmo Salar*)						Salmon (*Oncorhynchus sp.*)						
Sampling	Wild			Farmed			Wild ^x^			Farmed ^y^			
	*N*	mean ± SD	Range	*N*	mean ± SD	Range	*N*	mean ± SD	Range	*N*	mean ± SD	Range	Ref
2007–2008				* 20	0.49					* 12	0.22		[17]
2007–2008				* 2 × 10	0.38	0.37 × 0.38	* 2 × 10	0.054	<0.1–0.11				[18]
2011										* 9	0.18		[39]
2014	5	0.30 ± 0.03	0.28–0.36										-
2016 ^b^				3	nd								[19]
2016	5	0.22 ± 0.05	0.15–0.27										-
2018				12	0.78 ± 0.30	0.37–1.5							[18]

* Composite samples, ^b^ Publication year. ^x^ *O. gorbuscha* and *O. keta*; *O. sp.* ^y^ *O. mykiss*; ”-“ this study; nd = not detected.

## Supplementary Materials

The following are available online at https://www.mdpi.com/2072-6643/11/5/982/s1, Table S1: Content in salmon feed for the feeding trial, Table S2: Two-way ANOVA for the vitamin D content in the farmed salmon.
