# Supplementary material for: Vitamin D in Wild and Farmed Atlantic Salmon (Salmo Salar)—What Do We Know?
Source: Nutrients. 2019 Apr 29;11(5):982. doi: 10.3390/nu11050982 (PMC6566758; doi:10.3390/nu11050982)
Supplement: Supplementary file 1 [file nutrients-11-00982-s001.pdf]

Supplementary Material

S1. Content in salmon feed for feeding trial…………………………………………… 2
S2. Two-way ANOVA for vitamin D content in farmed salmon……………………..3

Table S1. Content in salmon feed for feeding trial.

Ingredients, %	Control
Fishmeal[1]	17.50
Fish protein concentrate[2]	2.50
Soy protein concéntrate[3]	17.00
Pea protein concentrate[4]	2.50
Wheat gluten[5]	10.25
Wheat meal[6]	12.00
Faba beans (low tannins)[7]	5.83
Fish oil[8]	27.60
Vitamin and mineral premix[9]	1.00
Binder[10]	0.30
Antioxidant[11]	0.20
Sodium propionate[12]	0.10
Monocalcium phosphate[13]	2.00
L-Histidine[14]	0.10
L-Lysine[15]	0.50
L-Tryptophan[16]	0.10
DL-Methionine[17]	0.30
L-Taurine[18]	0.22

[1] Fishmeal LT70 NORVIK: 70.6% crude protein (CP), 5.8% crude fat (CF), Sopropêche, France. [2] CPSP90: 84% CP, 9.6% CF, SOPROPÊCHE, France. [3] Soycomil P: 63% CP, 0.8% CF, ADM, The Netherlands. [4] Lysamine GPS: 86% CP, 1% CF, ROQUETTE Frères, France. [5] VITAL: 83.7% CP, 1.6% CF, ROQUETTE Frères, France. [6] Wheat meal: 10.2% CP, 1.2% CF, Casa Lanchinha, Portugal. [7] Faba beans: 28.5% CP; 1.2% CF, Ribeiro & Sousa Cereais, Portugal. [8] Sopropêche, France. [9] PREMIX Lda, Portugal: Vitamins (IU or mg/kg diet): DL-alpha tocopherol acetate, 100 mg; sodium menadione bisulphate, 25mg; retinyl acetate, 20000 IU; DL-cholecalciferol, 2000 IU; thiamin, 30mg; riboflavin, 30mg; pyridoxine, 20mg; cyanocobalamin, 0.1mg; nicotinic acid, 200mg; folic acid, 15mg; ascorbic acid, 500mg; inositol, 500mg; biotin, 3mg; calcium panthotenate, 100mg; choline chloride, 1000mg, betaine, 500mg. Minerals (g or mg/kg diet): copper sulphate, 9mg; ferric sulphate, 6mg; potassium iodide, 0.5mg; manganese oxide, 9.6mg; sodium selenite, 0.01mg; zinc sulphate,7.5mg; sodium chloride, 400mg; excipient wheat middling's. [10] Kieselguhr (natural zeolite), LIGRANA GmbH, Germany. [11] Paramega PX, Kemin Europe NV, Belgium. [12] Disproquímica, Portugal. [13] MCP: 22% P, 18% Ca, Fosfitalia, Italy. [14] L-Histidine 98%, Ajinomoto Eurolysine SAS, France. [15] L-Lysine HCl 99%: Ajinomoto Eurolysine SAS, France. [16] TrypAMINO 98%, Evonik Nutrition & Care GmbH, Germany. [17] DL-METHIONINE FOR AQUACULTURE 99%, EVONIK Nutrition & Care GmbH, Germany. [18] L-Taurine, ORFFA, The Netherlands.

All powder ingredients were mixed accordingly to the target formulation in a double-helix mixer (model 500L, TGC Extrusion, France) and ground (below 400 μm) in a micropulverizer hammer mill (model SH1, Hosokawa-Alpine, Germany). The control diet (pellet size: 7.0 mm) was manufactured with a twin-screw extruder (model BC45, Clextral, France) with a screw diameter of 55.5 mm. Extrusion conditions: feeder rate (77 kg/h), screw speed (247 rpm), water addition in barrel 1 (330 ml/min), temperature barrel 1 (32-34ºC), temperature barrel 3 (111-115°C). Extruded pellets were dried in a vibrating fluid bed dryer (model DR100, TGC Extrusion, France). After cooling, the various doses of vitamin D3 (ROVIMIX® D3 500; cholecalciferol at 500 000 IU per gram of product) were blended with the oil and applied post-extrusion to the pellets by vacuum coating (model PG-10VCLAB, Dinnissen, The Netherlands). Immediately after coating, diets were packed in sealed plastic buckets and shipped to Bantry, Ireland.

Table S2. Two-way ANOVA table for vitamin D content in farmed salmon.

Source of Variation	*SS*	*df*	*MS*	*F*	*P-value*	*F crit*
Weeks of feeding	11,42857	2	5,714285	5,832773	0,0054	3,190727
Vitamin D in feed	140,3214	3	46,77379	47,74367	1,91E-14	2,798061
Interaction	26,66382	6	4,443969	4,536117	0,001019	2,294601
Within	47,02492	48	0,979686			
Total	225,4387	59				
